# Stillbirth classification in population-based data and role of fetal growth restriction: the example of RECODE

**DOI:** 10.1186/1471-2393-13-182

**Published:** 2013-10-03

**Authors:** Anne Ego, Jennifer Zeitlin, Pierre Batailler, Séverine Cornec, Anne Fondeur, Marion Baran-Marszak, Pierre-Simon Jouk, Thierry Debillon, Christine Cans

**Affiliations:** 1 The RHEOP (Registre des Handicaps de l’Enfant et Observatoire Périnatal Isère, Savoie et Haute-Savoie), Grenoble, France; 2 INSERM, UMR S953, Epidemiological Research on Perinatal Health and Women's and Children's Health, Paris, France; 3 The THEMAS, UJF-Grenoble 1, Grenoble, France; 4 DMIS, Pavillon Taillefer, CHU Grenoble, CS 10217, 38043 Grenoble Cedex 9, France

**Keywords:** Stillbirths, Classification, Cause of death, Associated conditions, Small for gestational age, Fetal growth

## Abstract

**Background:**

Stillbirth classifications use various strategies to synthesise information associated with fetal demise with the aim of identifying key causes for the death. RECODE is a hierarchical classification of death-related conditions, which grants a major place to fetal growth restriction (FGR). Our objective was to explore how placement of FGR in the hierarchy affected results from the classification.

**Methods:**

In the Rhône-Alpes region, all stillbirths were recorded in a local registry from 2000 to 2010 in three districts (N = 969). Small for gestational age (SGA) was defined as a birthweight below the 10^th^ percentile. We applied RECODE and then modified the hierarchy, including FGR as the penultimate category (RECODE-R).

**Results:**

49.0% of stillbirths were SGA. From RECODE to RECODE-R, stillbirths attributable to FGR decreased from 38% to 14%, in favour of other related conditions. Nearly half of SGA stillbirths (49%) were reclassified. There was a non-significant tendency toward moderate SGA, singletons and full-term stillbirths to older mothers being reclassified.

**Conclusions:**

The position of FGR in hierarchical stillbirth classification has a major impact on the first condition associated with stillbirth. RECODE-R calls less attention to monitoring SGA fetuses but illustrates the diversity of death-related conditions for small fetuses.

## Background

Classifications of perinatal deaths are needed for health care policy, surveillance, international comparisons, clinical services, and research. There is a wide variety of these classifications in the literature, reflecting differences in criteria and available information for recording stillbirths and in existing health information systems over time and between countries [1,2]. Some of them include categories best suited for epidemiology and health care planning purposes, including risk factors such as small for gestational age (SGA) or twin pregnancy, while others aspire to provide information on the cause of death, focusing on specific clinical groups relevant to biomedical research questions [3].

After a substantial decrease of the stillbirth rate, by two-thirds from 1950 to 1975, related to prevention and treatment of infection and improved obstetric care, this decline has slowed or halted in high-income countries during the last few decades [4]. Authors of the Lancet’s Stillbirths Series in 2011 suggested that classification should be the first research priority in epidemiological measurement, and underline the need for “the optimum investigation protocol for stillbirth to identify causes and relevant conditions in terms of yield, utility and costs” in high-income countries. Most classifications consistently report up to two-thirds of fetal deaths as being unexplained or unknown [1]. Several factors contribute to increasing the number of unexplained or unknown cases, such as the design of the system itself or the lack of postmortem investigation.

The classification called RECODE (RElevant COndition at DEath) is intended to be used in a strictly hierarchical manner and designed to organize information on the clinical conditions associated with the death rather than why the death occurred [5]. This makes it possible to avoid a case-by-case analysis of the circumstances leading to the death and to apply the classification retrospectively to existing databases. Other strengths of this classification are that is has a clear hierarchical structure, is based on ICD codes, and enables 85% of stillbirth cases to be assigned a relevant condition. In 2009, RECODE was ranked third in the International Stillbirth Alliance out of six contemporary systems designed specifically for stillbirths: Amended Aberdeeen, Extended Wigglesworth, PSANZ-PDC, CODAC and Tulip [3]. They concluded that the best classifications collect all relevant information, use a hierarchical approach as a guide, but rely on expert opinions in order to preserve the relative importance of the narrative [6-8].

The RECODE classification grants significant importance to fetal growth restriction (FGR) relative to other clinical conditions. This is concordant with previous analyses of the pathophysiology of conditions underlying stillbirths [2]. This choice is also supported by the potential preventability of stillbirths associated with FGR [9]. However, the placement of FGR in the RECODE classification may override important information on other related conditions. For instance, when autopsy and placental examinations exist they provide information on placental pathology, which is a frequent antecedent of both FGR and stillbirth [10]. These anomalies are also part of a large group of clinical scenarios associated with maternal vascular disease and FGR [11,12].

The aim of this study was to test how the hierarchical ranking of FGR affected the classification of stillbirths in a large population-based registry in the Rhône-Alpes region from 2000 to 2010. We compared the RECODE classification with an alternative hierarchy, labelled RECODE-R in which FGR was only retained in the absence of other clinical conditions.

## Method

### Study design

The RHEOP (Registry of childhood handicaps and perinatal observatory) was created in 1988 in the Isère district in the Rhône-Alpes region of France. The area covered by the registry was enlarged to include two contiguous districts in 2005 (Savoie and Haute-Savoie). This registry includes all cases of childhood disability as well as all stillbirths to residents in these districts [13]. Its objective is to monitor the trends in stillbirth, to identify causes of death, and to improve the interpretation of trends in childhood disability by taking into consideration trends in stillbirths and pregnancy terminations. The three participating districts constitute a population-based sample of 30 000 births per year. The RHEOP registry uses the WHO definition of a stillbirth, i.e., “the birth of a baby with a birth weight of 500 g or 22 or more completed weeks of gestation who died before or during labor and birth” [14].

The RHEOP stillbirth register was approved by the French data protection authority Commission on Information Technology and Liberties (CNIL) (approval number 997086). This approval covers secondary analyses of these data.

Stillbirths are identified in maternity hospitals by several investigators, who are trained nurses, midwives or physicians. They complete a standardized form based on the medical record for each case, which contains maternal age, occupation and profession, medical history, complications of pregnancy, findings of prenatal screening, elective terminations of pregnancy, delivery mod, time of death, gestational age and birth weight, and placental examination or fetal autopsy when this exists. Fetal autopsy and/or placental examination were performed for 77.4% of the study sample. Secondarily, the investigators encode the information into ICD codes (10^th^ edition) up to two maternal and six fetal diagnoses.

For the purposes of the study, we excluded all elective pregnancy terminations. The database consisted of 1030 stillbirths weighing 500 g or more, or 22 or more completed weeks of gestation, distributed over 11 years from 2000 to 2010, corresponding to a stillbirth rate of 3.8 per 1000 total births.

### Definition of SGA

Because maternal weight, height and parity were not recorded, we were not able to define SGA by customized birth weight standards [15]. We used a previous French multicenter study intended to develop and evaluate customized birth weight curves suitable for France [16]. We defined SGA using the 10^th^ centile of sex differentiate norms according to Hadlock’s formula for fetal growth curves, fitted to birth weights registered in the French Perinatal Survey in 1998 [17,18]. Severe SGA babies (below the 3^rd^ percentile) were distinguished from moderate SGA babies (3^rd^–10^th^ percentile). This information encoded in ICD code was added retrospectively whether or not this diagnosis was mentioned in the patient’s case notes.

We used the term “SGA” to refer to fetuses with a birthweight under the 10^th^ percentile, whereas the term “FGR” refers to the condition retained from the classification.

### Classification program

The RECODE classification contains 9 main categories from A (fetal conditions) to I (unclassified), each of them divided into several subgroups, totalling 37 subcategories [5]. These categories are anatomically ranged from fetal diseases to external maternal injury, and contained a variety of fetal and maternal diseases called conditions. Among the clinical conditions provided for each case, the primary condition is the first on the hierarchical list that is applicable to a case. A secondary condition can be defined on this list. FGR is the last subcategory in category A corresponding to fetal conditions. Unexplained cases are divided into two subcategories in RECODE: either cases with irrelevant conditions despite information or cases lacking available information.

For registry data to be used retrospectively, each clinical condition converted to the ICD code had to be assigned a subcategory. We sought the help of RECODE’s authors for matching each distinct maternal or fetal ICD code in the database with a subcategory. Forty-eight per cent of the ICDs codes in our database (174/360) had already been mapped. Among the blocks related to the perinatal period “O” (pregnancy, childbirth and the puerperium), “P” (certain conditions originating in the perinatal period), and to congenital malformations, deformations and chromosomal abnormalities “Q”, this rate was 64%. The remaining codes were more often codes assigned to maternal disease or conditions irrelevant to the death, or to different extensions of codes previously mapped.

The next step consisted in repeating a merging procedure between the main database and two additional files containing maternal and fetal ICD codes and their associated subcategory, for each of the eight potential diagnoses per case. The RECODE hierarchical rules were applied twice to select the first and the second relevant conditions. Lastly, the alternative hierarchy RECODE-R was tested. RECODE-R consisted in moving FGR down just above the unexplained cases, so that growth failure was retained only in the absence of all other conditions.

### Analysis

Stillbirths with missing data on gestational age, birth weight or sex were excluded. We described our population study and the results of the classification in the whole sample and for SGA stillbirths. Cases, for whom the first condition moved from RECODE to RECODE-R, were designated as “reclassified.” Reclassified SGA stillbirths were compared to SGA cases that were not reclassified.

Statistical analysis was performed using Intercooled STATA (Version 10, Stata Corporation, College Station, TX, USA); χ^2^ tests were used for qualitative variables and Student’s test for continuous variables. *P*-values less than 0.05 were considered statistically significant.

## Results

During the study period, 1030 stillbirths were recorded, and 61 (5.9%) were excluded due to missing data on gestational age (*n* = 1), birth weight (*n* = 42), sex (*n* = 24), gestational age below 22 weeks (*n* = 1) or gender ambiguity (*n* = 5). They were more often preterm fetal deaths (88.3%, *p* = 0.001) and multiple pregnancies (26.3%, *p* = 0.001). The final sample contained 969 stillbirths.

Table 1 presents the main characteristics of the sample. Antepartum and intrapartum deaths represented respectively, 81.6 and 15.0% of the cases, and 26.8% of the cohort were full-term stillbirths. Maternal age was below 25 and above 35 years old in 17.8 and 24.7%, respectively. Twelve per cent were twin pregnancies. The rate of SGA stillbirths was 49.0%, and most of them had a birth weight below the 3^rd^ centile (39.2%).

**Table 1:** Characteristics of stillbirths in the RHEOP registry, 2000–2010

**Characteristics**		**Total (n = 969)**	
		***n***	**%**
		mean ± SD	
Maternal age (years)	<25	172	17.8
	25-29	276	28.5
	30-34	278	28.7
	≥35	239	24.7
	Missing	4	0.4
Gestational age (completed weeks)		30.7 ±6.4	
Gestational age (completed weeks)	22–28	406	41.9
	29–36	303	31.3
	37+	260	26.8
Birth weight (grams)		1552 ±1114	
Birth weight percentile	≥10th	494	51.0
	3rd–10th	95	9.8
	<3rd	380	39.2
Gender	Male	516	53.3
	Female	453	46.7
Multiple birth	Yes	115	11.9
	No	823	84.9
	Missing	31	3.2
Time of death	Intrapartum	145	15.0
	Antepartum	791	81.6
	Missing	33	3.4

Table 2 shows the distribution of RECODE and RECODE-R categories and subcategories for all stillbirths and for all SGA stillbirths (the group non-SGA stillbirths only is not displayed in the table). Category A was composed of lethal congenital anomalies (A1), infection (A2), non-immune hydrops (A3), iso-immunization (A4), feto-maternal haemorrhage (A5), twin–twin transfusion (A6) and FGR (A7), and accounted for 58.7% of conditions retained in the total sample with RECODE. Its largest subcategory was A7 FGR (38.2%). The next three main categories were umbilical cord (B), placenta (C) and amniotic fluid (D), accounting for 6.7, 12.3, and 5.2%, respectively. Each of the other categories (uterus E, mother F, intrapartum G, trauma H) did not exceed 1.3%.

**Table 2:** RECODE and RECODE-R classifications among the whole sample and SGA stillbirths

**Primary relevant condition of death†**	**RECODE**				**RECODE-R**			
	**Total (*n*** **= 969)**		**SGA (*n*** **= 475)**		**Total (*n*** **= 969)**		**SGA (*n*** **= 475)**	
*Categories* and subcategories	*n*	%	*n*	%	*n*	%	*n*	%
*A-Foetus*	*569*	*58.7*	*475*	*100.0*	*335*	*34.6*	*241*	*50.7*
A1-Lethal congenital anomaly	142	14.7	83	17.5	142	14.7	83	17.5
A2-Infection	33	3.4	12	2.5	33	3.4	12	2.5
A3-Non-immune hydrops	13	1.3	3	0.6	13	1.3	3	0.6
A5-Foetomaternal haemorrhage	11	1.1	7	1.5	11	1.1	7	1.5
A7-Fetal growth restriction	370	38.2	370	77.9	136	14.0	*136*	*28.6*
*B-Umbilical cord*	*65*	*6.7*			*116*	*12.0*	*51*	*10.7*
B1-Prolapse	4	0.4			5	0.5	1	0.2
B2-Constricting loop or knot	54	5.6			97	10.0	43	9.0
B4-Umbilical cord - Other	7	0.7			14	1.4	7	1.5
*C-Placenta*	*119*	*12.3*			*240*	*24.8*	*121*	*25.5*
C1-Placenta abruptio	68	7.0			103	10.6	35	7.4
C2-Placenta praevia	1	0.1			2	0.2	1	0.2
C3-Vasa praevia	4	0.4			5	0.5	1	0.2
C4-Placental insufficiency	33	3.4			96	9.9	63	13.3
C5-Placenta - Other	13	1.3			34	3.5	21	4.4
*D-Amniotic fluid*	*50*	*5.2*			*100*	*10.3*	*50*	*10.5*
D1-Chorioamnionitis	36	3.7			55	5.7	19	4.0
D2-Oligohydramnios	4	0.4			27	2.8	23	4.8
D3-Polyhydramnios	7	0.7			11	1.1	4	0.8
D4-Amniotic fluid - Other	3	0.3			7	0.7	4	0.8
*E-Uterus*	*4*	*0.4*			*5*	*0.5*	*1*	*0.2*
E2-Anomalies	4	0.4			5	0.5	1	0.2
*F-Mother*	*13*	*1.3*			*22*	*2.3*	*9*	*1.9*
F1-Diabetes	2	0.2			2	0.2		
F4-Hypertensive diseases in pregnancy					1	0.1	1	0.2
F6-Cholestasis	1	0.1			2	0.2	1	0.2
F7-Drug misuse					1	0.1	1	0.2
F8-Maternal - Other	10	1.0			16	1.7	6	1.3
*G-Intrapartum*	*12*	*1.2*			*14*	*1.4*	*2*	*0.4*
G1-Asphyxia	10	1.0			12	1.2	2	0.4
G2-Birth trauma	2	0.2			2	0.2		
*H-Trauma*	*2*	*0.2*			*2*	*0.2*		
H1-External trauma	2	0.2			2	0.2		
*I-Unclassified*	*135*	*13.9*			*135*	*13.9*		
I1-No relevant condition identified	102	10.5			102	10.5		
I2-No information available	33	3.4			33	3.4		

† Subcategories with results equal to zero were not mentioned.

**Figure 1:**
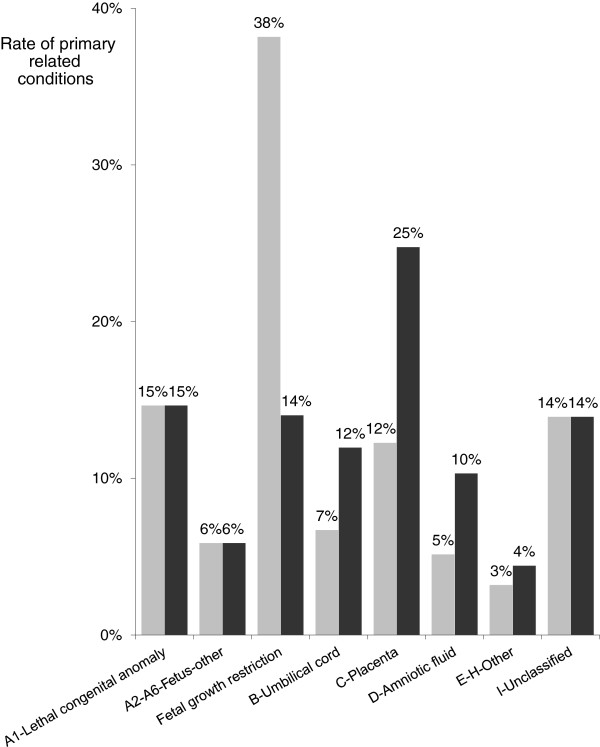
**Classification of stillbirths according to RECODE (gray) and RECODE-R (black) (*n*** **= 969).**

The main changes from RECODE to RECODE-R in the overall sample are also represented in Figure 1. According to the frequencies in the category A subcategories, we distinguished lethal congenital anomalies (A1) from “fetus-others” corresponding to A2–A6, and FGR (A7). Inversely, categories E–H were combined. From RECODE to RECODE-R, category A decreased substantially from 58.7% to 34.6%, its largest subcategory being now lethal congenital anomalies (14.7%) just before FGR (14.0%). This change increased the numbers of cases in the umbilical cord, placenta and amniotic fluid categories, which nearly doubled to 12.0, 24.8 and 10.3%, respectively. For the categories assigned to uterus, mother, intrapartum event, and trauma, only a slight increase (+1.2%) was observed.

Considering the hierarchical rule of RECODE, all SGA stillbirths were classified in category A, and FGR was retained in 77.9% (Table 2). The distribution of death conditions was radically different among non-SGA stillbirths: category A accounted for only 19% (*n* = 94), including 11.9% (*n* = 59) lethal congenital anomalies, and the main categories B–H were more frequently assigned. According to RECODE, unclassified deaths (*n* = 135, 13.9% of the whole sample) come exclusively from non-SGA stillbirths, and accounted for nearly one-third of them (27.3%).

Moving FGR down in the RECODE-R hierarchy had no impact on SGA births initially assigned to the subcategories A1–A6 (*n* = 105, 22.1%) (Table 2). By the design of RECODE-R, only stillbirths affected by growth failure and other diseases were redistributed. These 234 cases accounted for 24.1% of the whole sample and 49.3% of all SGA stillbirths. Only 136 SGA births (28.6%) remained classified as FGR. The new related conditions assigned to SGA stillbirths were placental insufficiency (13.3%), constricting loop or knot (9.0%) and placenta abruptio (7.4%).

Table 3 compares the characteristics of reclassified (*n* = 234) and non-reclassified (*n* = 136) stillbirths among the 370 stillbirths initially classified as FGR according to RECODE-R. The changes were independent of gestational age, sex, birth weight ratio, maternal age and time of death. There was a non-significant tendency for full-term babies (*p* = 0.06), stillbirths to older women (*p* = 0.16), singletons (*p* = 0.07) and moderate SGA babies (3^rd^–10^th^ centile) (*p* = 0.11) to be reclassified.

**Table 3:** **Characteristics of SGA stillbirths previously classified FGR with RECODE according their reclassification with RECODE-R (*n*** **= 370)**

**Stillbirth**	**Reclassified (*n*** **= 234)**		**Non-reclassified**		**p**
**characteristics**			**(*n*** **= 136)**		
	**n**	**%**	**n**	**%**	
	**mean ± sd**		**mean ± sd**		
Maternal age (years)	30.1	5.6	29.1	5.9	NS
Maternal age (years)					NS
<25y	44	18.8	28	20.6	
25–29y	65	27.8	44	32.4	
30–34y	69	29.5	44	32.4	
≥35y	56	23.9	19	14.0	
Unknown			1	0.7	
Gestational age (completed weeks)	30.3	±6.1	29.1	±5.7	NS
Gestational age (completed weeks)					NS
22–28w	100	42.7	71	52.2	
29–36w	79	33.9	42	30.9	
full-term	55	23.5	23	16.9	
Birth weight (grams)	1157	±867	1012	±791	NS
Birth weight percentile					NS
<3rd	176	75.2	112	82.4	
3rd–10th	58	24.8	24	17.8	
Gender					NS
Male	123	52.6	73	53.7	
Female	111	47.4	63	46.3	
Multiple pregnancy					NS
Yes	27	11.5	24	17.7	
No	202	86.3	105	77.2	
Unknown	5	2.1	7	5.1	
Time of death					NS
Intrapartum	24	10.3	12	8.8	
Antepartum	203	86.8	119	87.5	
Unknown	7	3.0	5	3.7	

NS Not Significant (*p* > 0.05).

## Discussion

We tested how the RECODE stillbirth classification performed in a retrospective analysis of a large population-based database of stillbirths. By moving FGR down in the RECODE hierarchy, so that low birthweight for gestational age was retained only in the absence of other conditions, the proportion of stillbirths assigned to the FGR category decreased from 38.2 to 14.0%. Related conditions of the umbilical cord, placenta and amniotic fluid were highlighted and selected in nearly half of the cases. In particular, with RECODE-R one stillbirth in four is assigned to the category of placental conditions. For SGA babies without congenital malformations or fetal abnormalities, these outcomes seemed to fit the mechanisms of death more closely and illustrate their diversity.

Surveillance of stillbirths in a population is an important epidemiological aim of a registry. There is a need for standardised classifications to improve our understanding of these events and how they evolve. For each death, a number of conditions are often observed that may have contributed to the death and the synthesis and organization of this information presents a challenge. We took the pragmatic point of view adopted by Gardosi et al. and demonstrated the feasibility of a RECODE hierarchical computer-based programme. Froen et al. distinguishes cause of death and associated conditions of death which only “contribute in explaining the circumstances of death in a significant proportion of deaths” [7]. From a clinical point of view, this approach may be frustrating compared to a case-by-case perinatal audit [7,8,19]. But this strategy is less time-consuming, retrospectively usable, suitable in an exhaustive and long-standing data collection, and avoids inconsistent identification of cause of death between investigators, countries or study periods. Its main drawback, however, is that it follows a pre-established hierarchy, regardless of whether another condition was evidently a more significant contributor.

The ICD was developed to allow the systematic coding, analysis, interpretation and comparison of morbidity and mortality, and worldwide estimates of stillbirths rate are often provided by these routinely collected data [20]. Recent classifications developed in high-income countries give priority to exhaustive individual analysis, even though some of them ensure compatibility with ICD [7]. The NICE and RECODE classifications are probably unique in using a strictly hierarchical and computerized method applied to ICD codes [21,22]. This approach is consistent with recent recommendations of the authors of The Lancet’s Stillbirths series, who advocate a consensus “on a limited number of programmatically relevant, comparable causal categories,… that can be linked to complex classification systems and ICD codes” [23]. This linkage may be improved if mapping could be extended to all ICD codes through a multi-disciplinary action in order to insure consensus on subcategory definitions. Indeed, not all our ICD codes were included in the initial West Midlands algorithm, suggesting that the choice of ICD codes for maternal and fetal conditions may differ by setting. Furthermore, better classifications could be developed if some of the limitations inherent to using ICD codes for the classification of stillbirths are modified in the revision of ICD-11 [3,22].

There are a few examples of the RECODE classification system in population-based samples. Our rate of unexplained cases was close to the West Midlands cohort of 2625 stillbirths, the Dutch sample of 485 antepartum singleton stillbirths, or the Italian sample of 154 stillbirths (16.0, 14.2 and 14.3%, respectively) [5,12,19]. Like Gardosi et al., we reported 15% lethal congenital anomalies, but our stillbirths classified as FGR (A7) was slightly lower (38.2% versus 43.0%). In the two other case series, the authors found only 30.3% and 16.9% FGR [12,19]. These differences could be due to population selection and most probably to different definitions of SGA births. In particular we were unable to use customized norms which require data on maternal height and weight. This adjustment strengthens the association between SGA and maternal and fetal complications, and the rate of SGA stillbirths was probably slightly underestimated in our study [24].

In our alternative RECODE-R hierarchy of classification, we considered SGA as a common modifier of other underlying maternal and fetal conditions, but not as a specific condition in itself, unless SGA was isolated. In the six classification systems for stillbirth analyzed by Flenady et al., RECODE is the only one with FGR classified as a specific condition [3]. Four of them do not mention FGR, and isolated FGR is put with unexplained cases at the bottom of the list [6,7,25-27]. The PSANZ (Perinatal Society of Australia and New Zealand) classification ranks FGR 8^th^ of 11 categories and placental histology defines the subcategories, resulting in a FGR rate of 3.2% in a recent analysis in New South Wales from 2002 to 2004 [6,28].

The impact of RECODE-R concerns SGA stillbirths associated with various conditions except fetal conditions. Nevertheless, the main characteristics of reclassified SGA stillbirths did not differ from those of non-reclassified SGA stillbirths. We only found a tendency for full-term, singletons, moderate SGA stillbirths, and stillbirths to mothers aged 35 years or more to be more often reclassified. Several explanations are plausible. Due to specific fetal anomalies, multiple pregnancies are more likely to stay in one of the group A subcategories. The reason that stillbirths to older mothers presented placental, umbilical or maternal conditions more often, and consequently were reclassified more often, may be related to a higher frequency of maternal complications with advanced maternal age. The mechanisms for full-term stillbirths is less clear especially as late stillbirths are those that are more likely to remain unexplained [29,30]. On the other hand, post-mortem investigations could be performed more often for full-term stillbirths, so that this information is highlighted. The mild severity of growth failure among full-term versus preterm stillbirths had already been described [31]. Finally the fact that severe compared to moderate SGA stillbirths stay preferentially in the FGR category might be a reasonable argument for using RECODE-R. The impact of RECODE-R on the classification of SGA stillbirths according to their characteristics, and the hypothesized mechanisms should be confirmed in larger studies.

## Conclusions

Monitoring stillbirth rates and capturing the reality of primary clinical conditions associated with fetal death remains an ambitious challenge. Using a hierarchical system within a classification requires defining priorities among the circumstances of death; this strategy is a complementary approach to the perinatal audit designed to identify cause of death. RECODE underlines the frequency of growth failure among stillbirths and the importance of improving prenatal detection of FGR. In contrast, RECODE-R may be closer to etiological mechanisms leading to death and supports the use of post-mortem investigations. Given that the selection of a classification leads to important differences in the clinical conditions which are underscored; these choices should be made explicit and justified with respect to the objective of the analyses.

## Abbreviations

SGA: Small for gestational age; FGR: Fetal growth restriction.

## Competing interests

The authors declare that they have no competing interests.

## Authors’ contributions

AE and JZ have been involved in conception and design, analysis and interpretation of data, and draft of the manuscript. PB has been involved in analysing the data and drafting the manuscript. CC, PSJ and TD revised it for important intellectual content and gave final approval of the version to be published. SC, AF and MBM contributed to acquisition of data. All authors read and approved the final manuscript.

## Acknowledgements

This work was supported by the Institut National de la Santé et de la Recherche Médicale (INSERM), the Institut National de Veille Sanitaire (INVS), and the Conseils Généraux of the districts of Isère, Savoie and Haute-Savoie. We particularly appreciated the experience and the help of Prof. Jason Gardosi for encoding the different RECODE conditions. We are also grateful to all the RHEOP members, especially Claire Ambrico, Magalie Piret and Catherine Tronc, for their contribution in data collection, completeness and quality control.

## Pre-publication history

The pre-publication history for this paper can be accessed here:

http://www.biomedcentral.com/1471-2393/13/182/prepub

## References

[B1] Gordijn SJ Korteweg FJ Erwich JJ Holm JP van Diem MT Bergman KA Timmer A A multilayered approach for the analysis of perinatal mortality using different classification systems Eur J Obstet Gynecol Reprod Biol 2009 144 2 99 104 10.1016/j.ejogrb.2009.01.012 19272694

[B2] Reddy UM Goldenberg R Silver R Smith GC Pauli RM Wapner RJ Gardosi J Pinar H Grafe M Kupferminc M Stillbirth classification–developing an international consensus for research: executive summary of a National Institute of Child Health and Human Development workshop Obstet Gynecol 2009 114 4 901 914 10.1097/AOG.0b013e3181b8f6e4 19888051 PMC2792738

[B3] Flenady V Froen JF Pinar H Torabi R Saastad E Guyon G Russell L Charles A Harrison C Chauke L An evaluation of classification systems for stillbirth BMC Pregnancy Childbirth 2009 9 24 10.1186/1471-2393-9-24 19538759 PMC2706223

[B4] Flenady V Middleton P Smith GC Duke W Erwich JJ Khong TY Neilson J Ezzati M Koopmans L Ellwood D Stillbirths: the way forward in high-income countries Lancet 2011 377 9778 1703 1717 10.1016/S0140-6736(11)60064-0 21496907

[B5] Gardosi J Kady SM McGeown P Francis A Tonks A Classification of stillbirth by relevant condition at death (ReCoDe): population based cohort study BMJ 2005 331 7525 1113 1117 10.1136/bmj.38629.587639.7C 16236774 PMC1283273

[B6] Chan A King JF Flenady V Haslam RH Tudehope DI Classification of perinatal deaths: development of the Australian and New Zealand classifications J Paediatr Child Health 2004 40 7 340 347 10.1111/j.1440-1754.2004.00398.x 15228558

[B7] Froen JF Pinar H Flenady V Bahrin S Charles A Chauke L Day K Duke CW Facchinetti F Fretts RC Causes of death and associated conditions (Codac): a utilitarian approach to the classification of perinatal deaths BMC Pregnancy Childbirth 2009 9 22 10.1186/1471-2393-9-22 19515228 PMC2706222

[B8] Dudley DJ Goldenberg R Conway D Silver RM Saade GR Varner MW Pinar H Coustan D Bukowski R Stoll B A new system for determining the causes of stillbirth Obstet Gynecol 2010 116 2 254 260 10.1097/AOG.0b013e3181e7d975 20664383 PMC3832680

[B9] Gardosi J Madurasinghe V Williams M Malik A Francis A Maternal and fetal risk factors for stillbirth: population based study BMJ 2013 346 f108 10.1136/bmj.f108 23349424 PMC3554866

[B10] Froen JF Gardosi JO Thurmann A Francis A Stray-Pedersen B Restricted fetal growth in sudden intrauterine unexplained death Acta Obstet Gynecol Scand 2004 83 9 801 807 15315590 10.1111/j.0001-6349.2004.00602.x

[B11] Kidron D Bernheim J Aviram R Placental findings contributing to fetal death, a study of 120 stillbirths between 23 and 40 weeks gestation Placenta 2009 30 8 700 704 10.1016/j.placenta.2009.05.009 19535137

[B12] Korteweg FJ Gordijn SJ Timmer A Holm JP Ravise JM Erwich JJ A placental cause of intra-uterine fetal death depends on the perinatal mortality classification system used Placenta 2008 29 1 71 80 10.1016/j.placenta.2007.07.003 17963842

[B13] Rey S Hoffmann P Arnould P Jouk PS Tronc C Cans C Tendances et caractéristiques de la mortinatalité dans trois départements alpins (l’Isère, la Savoie et la Haute-Savoie) Revue de médecine périnatale 2009 1 4 200 207

[B14] WHO International statistical classification of diseases and related health problems. Tenth Revision 1993 2 Geneva, Switzerland: World Health Organization 129

[B15] Gardosi J Mongelli M Wilcox M Chang A An adjustable fetal weight standard Ultrasound Obstet Gynecol 1995 6 3 168 174 10.1046/j.1469-0705.1995.06030168.x 8521065

[B16] Ego A Subtil D Grange G Thiebaugeorges O Senat MV Vayssiere C Zeitlin J Customized versus population-based birth weight standards for identifying growth restricted infants: a French multicenter study Am J Obstet Gynecol 2006 194 4 1042 1049 10.1016/j.ajog.2005.10.816 16580294

[B17] Blondel B Norton J du Mazaubrun C Breart G Development of the main indicators of perinatal health in metropolitan France between 1995 and 1998. Results of the national perinatal survey Journal de gynecologie, obstetrique et biologie de la reproduction 2001 30 6 552 564 11883022

[B18] Hadlock FP Harrist RB Martinez-Poyer J In utero analysis of fetal growth: a sonographic weight standard Radiology 1991 181 1 129 133 1887021 10.1148/radiology.181.1.1887021

[B19] Vergani P Cozzolino S Pozzi E Cuttin MS Greco M Ornaghi S Lucchini V Identifying the causes of stillbirth: a comparison of four classification systems Am J Obstet Gynecol 2008 199 3 e311 314 10.1016/j.ajog.2008.06.098 18771999

[B20] Cousens S Blencowe H Stanton C Chou D Ahmed S Steinhardt L Creanga AA Tuncalp O Balsara ZP Gupta S National, regional, and worldwide estimates of stillbirth rates in 2009 with trends since 1995: a systematic analysis Lancet 2011 377 9774 1319 1330 10.1016/S0140-6736(10)62310-0 21496917

[B21] Winbo IG Serenius FH Dahlquist GG Kallen BA NICE, a new cause of death classification for stillbirths and neonatal deaths. Neonatal and Intrauterine Death Classification according to Etiology Int J Epidemiol 1998 27 3 499 504 10.1093/ije/27.3.499 9698143

[B22] Froen JF Gordijn SJ Abdel-Aleem H Bergsjo P Betran A Duke CW Fauveau V Flenady V Hinderaker SG Hofmeyr GJ Making stillbirths count, making numbers talk - issues in data collection for stillbirths BMC Pregnancy Childbirth 2009 9 58 10.1186/1471-2393-9-58 20017922 PMC2805601

[B23] Lawn JE Blencowe H Pattinson R Cousens S Kumar R Ibiebele I Gardosi J Day LT Stanton C: S Where? When? Why? How to make the data count? Lancet 2011 377 9775 1448 1463 10.1016/S0140-6736(10)62187-3 21496911

[B24] Gardosi J Figueras F Clausson B Francis A The customised growth potential: an international research tool to study the epidemiology of fetal growth Paediatr Perinat Epidemiol 2011 25 1 2 10 10.1111/j.1365-3016.2010.01166.x 21133964

[B25] Cole SK Hey EN Thomson AM Classifying perinatal death: an obstetric approach Br J Obstet Gynaecol 1986 93 12 1204 1212 10.1111/j.1471-0528.1986.tb07853.x 3801350

[B26] Korteweg FJ Gordijn SJ Timmer A Erwich JJ Bergman KA Bouman K Ravise JM Heringa MP Holm JP The Tulip classification of perinatal mortality: introduction and multidisciplinary inter-rater agreement Bjog 2006 113 4 393 401 10.1111/j.1471-0528.2006.00881.x 16553651

[B27] Wigglesworth JS Monitoring perinatal mortality. A pathophysiological approach Lancet 1980 2 8196 684 686 6106794 10.1016/s0140-6736(80)92717-8

[B28] Gordon A Jeffery HE Classification and description of stillbirths in New South Wales, 2002–2004 Med J Aust 2008 188 11 645 648 18513173 10.5694/j.1326-5377.2008.tb01822.x

[B29] Froen JF Arnestad M Frey K Vege A Saugstad OD Stray-Pedersen B Risk factors for sudden intrauterine unexplained death: epidemiologic characteristics of singleton cases in Oslo, Norway, 1986–1995 Am J Obstet Gynecol 2001 184 4 694 702 10.1067/mob.2001.110697 11262474

[B30] Huang DY Usher RH Kramer MS Yang H Morin L Fretts RC Determinants of unexplained antepartum fetal deaths Obstet Gynecol 2000 95 2 215 221 10.1016/S0029-7844(99)00536-0 10674582

[B31] Gardosi J Systematic reviews: insufficient evidence on which to base medicine Br J Obstet Gynaecol 1998 105 1 1 5 10.1111/j.1471-0528.1998.tb09339.x 9442151

